# BrabA.11339.a: anomalous diffraction and ligand binding guide towards the elucidation of the function of a ‘putative β-lactamase-like protein’ from *Brucella melitensis*

**DOI:** 10.1107/S1744309111010220

**Published:** 2011-08-16

**Authors:** Jan Abendroth, Banumathi Sankaran, Thomas E. Edwards, Anna S. Gardberg, Shellie Dieterich, Janhavi Bhandari, Alberto J. Napuli, Wesley C. Van Voorhis, Bart L. Staker, Peter J. Myler, Lance J. Stewart

**Affiliations:** a Seattle Structural Genomics Center for Infectious Disease (SSGCID), USA; b Emerald BioStructures Inc., 7869 NE Day Road West, Bainbridge Island, WA 98110, USA; c Berkeley Center for Structural Biology, Lawrence Berkeley Laboratory, 1 Cyclotron Road, Building 6R2100, Berkeley, CA 94720, USA; d School of Medicine, Department of Allergy and Infectious Diseases, University of Washington, Seattle, WA 98195, USA; e Seattle Biomedical Research Institute, 307 Westlake Avenue North, Suite 500, Seattle, WA 98109, USA

**Keywords:** Seattle Structural Genomics Center for Infectious Disease, iodide, SAD phasing, anomalous diffraction, *Brucella melitensis*, lactamase, Phn

## Abstract

The crystal structure of a β-lactamase-like protein from *Brucella melitensis* was initially solved by SAD phasing from an in-house data set collected on a crystal soaked with iodide. A high-resolution data set was collected at a synchroton at the Se edge wavelength, which also provided an independent source of phasing using a small anomalous signal from metal ions in the active site. Comparisons of anomalous peak heights at various wavelengths allowed the identification of the active-site metal ions as manganese. In the native data set a partially occupied GMP could be identified. When co-crystallized with AMPPNP or GMPPNP, clear density for the hydrolyzed analogs was observed, providing hints to the function of the protein.

## Abstract 2

The structure of a β-lactamase-like protein from *B. melitensis* was solved independently using two data sets with anomalous signal. Anomalous Fourier maps could confirm the identity of two metal ions in the active site. AMP-bound and GMP-bound structures provide hints to the possible function of the protein.

## 1. Introduction

The mission of the Seattle Structural Genomics Center for Infectious Disease (SSGCID) is to provide the scientific community with a library of crystal structures of proteins from infectious organisms that are potential drug targets. This collection of structures is hoped to promote structure-guided drug design against National Institutes for Allergy and Infectious Disease (NIAID) class A, B and C infectious disease organisms. The target organisms are divided between SSGCID and its sister organization the Center for Structural Genomics of Infectious Diseases (CSGID). Both centers provide plasmids (BEI repository), protein samples (SSGCID), coordinates and structure factors (Protein Data Bank), and raw diffraction images (http://www.csgid.org/csgid/pages/diffraction_images) to the community immediately after completion of a target.

*Brucella melitensis* is a Gram-negative bacterial pathogen and one of the several *Brucella* species identified to cause brucellosis (Moreno & Moriyon, 2002 ▶). Brucellosis affects mostly goats, sheep and cattle, causing reproductive diseases such as abortions and stillbirths, hence the popular name ‘contagious abortion’. The pathogen can be transmitted to humans *via* contact with infected animals or the con­sumption of unpasteurized milk or cheese (Young, 1995 ▶). In humans the disease was initially called ‘Malta fever’ or ‘undulating fever’ and is multi-systemic, with nonspecific symptoms such as fever, chills, dementia, fatigue, headaches, nausea, vomiting, muscular and bodily pain. Regions with significant impact of brucellosis are parts of the Mediterranean, Asia, Africa and Latin America (Corbel, 1997 ▶). Infections can be successfully treated with antimicrobial agents. The pathobiology of *Brucella* is unique owing to the absence of classical virulence factors (Moreno & Moriyon, 2002 ▶).

Here, we report the high-resolution structure of BrabA.11339.a, a structural genomics target that entered the pipeline as a ‘β-lactamase-like protein’. β-Lactamases break down β-lactam antibiotics such as penicillin and lead to the resistance of pathogens to antibiotics. There are four classes of β-lactamase: classes A–D. While classes A, C and D have an active site-serine, class B metallo-β-­lactamases have a binuclear metal center in the active site that activates a water. With high-resolution data including anomalous information at various wavelengths, we could obtain a detailed description of the active site of BrabA.11339.a and an identification of the active-site metals. Ligand-bound structures lead us towards an understanding of the function of this protein.

## 2. Materials and methods

### 2.1. Cloning, expression and purification

The gene for BrabA.11339.a from *B. melitensis* biovar Abortus 2308 (NCBI YP_414422.1; locus BAB1_1016; UniProt Q2YQ74) spanning the full-length protein from residues 2–272 was cloned into a pAVA0421 vector using ligation-independent cloning (LIC; Aslanidis & de Jong, 1990 ▶; Mehlin *et al.*, 2006 ▶). The vector encodes an N-­terminal histidine-affinity tag followed by the substrate sequence for 3C protease; the entire tag sequence is MAHHHHHHMGTLEAQTQ′GPGS, where ′ indicates the cleavage site.

The protein was expressed in *Escherichia coli* using BL21 (DE3) R3 Rosetta cells and autoinduction medium (Studier, 2005 ▶) in a LEX Bioreactor. Starter cultures of LB broth with appropriate antibiotics were grown for ∼18 h at 310 K. ZYP-5052 auto-induction medium was freshly prepared as per Studier’s published protocol (Studier, 2005 ▶). Antibiotics were added to 2 l bottles of sterile auto-induction medium. The bottles were inoculated with all of the overnight culture. Inoculated bottles were then placed into a LEX bio­reactor. Cultures were grown for ∼24 h at 298 K; the temperature was reduced to 288 K and the cultures were grown for a further ∼60 h. To harvest, the medium was centrifuged at 4000*g* for 20 min at 277 K. The cell paste was flash-frozen in liquid nitrogen and stored at 193 K.

The frozen cells were resuspended in lysis buffer (25 m*M* HEPES pH 7.0, 500 m*M* NaCl, 5% glycerol, 30 m*M* imidazole, 0.025% sodium azide, 0.5% CHAPS, 10 m*M* MgCl_2_, 1 m*M* TCEP, 250 ng ml^−1^ AEBSF and 0.05 µg ml^−1^ lysozyme). The resuspended cell pellet was dis­rupted on ice for 30 min with a Virtis sonicator (408912) set to 100 W power with alternating cycles of 15 s pulse-on and 15 s pulse-off. The cell debris was incubated with 20 µl Benzonase nuclease (25 units ml^−1^) at room temperature for 45 min and clarified by centrifugation using a Sorvall SLA-1500 centrifuge at 14 000 rev min^−1^ for 75 min at 277 K. The protein was purified from the clarified cell lysate by immobilized metal-affinity chromatography using a 5 ml HisTrap FF column (GE Healthcare) equilibrated with binding buffer (25 m*M* HEPES pH 7.0, 500 m*M* NaCl, 5% glycerol, 30 m*M* imidazole, 0.025% sodium azide, 1 m*M* TCEP). The recombinant protein was eluted with 250 m*M* imidazole. The hexahistidine tag was removed by overnight incubation with 3C protease. The sample was then passed over HisTrap resin in a gravity-flow column. The tagless protein was collected in the flowthrough. It was further purified by size-exclusion chromatography (SEC) using a HiLoad 26/60 Superdex 75 column (GE Healthcare) equilibrated with SEC buffer (25 m*M* HEPES pH 7.0, 500 m*M* NaCl, 2 m*M* DTT, 0.025% sodium azide and 5% glycerol). The protein eluted as a single peak. The peak fractions were pooled and concentrated to 19.6 mg ml^−1^ using an Amicon Ultra centrifugal filter with a 10 kDa cutoff (Millipore). The concentrated sample was flash-frozen in liquid nitrogen and stored at 193 K.

### 2.2. Crystallization and X-ray data collection

For the target BrabA.11339.a at a protein concentration of 19.6 mg ml^−1^, two sparse-matrix screens were set up following the strategy of Newman *et al.* (2005 ▶): JCSG+ (Emerald Bio­Systems) and PACT (Molecular Dimensions). 0.4 µl protein solution was mixed with 0.4 µl well solution and equilibrated against 100 µl reservoir using 96-well Compact Jr crystallization plates (Emerald Bio­Systems). Crystals suitable for diffraction studies were obtained under two very similar conditions from the PACT screen: G9 (200 m*M* sodium/potassium tartrate, 100 m*M* Bis-Tris propane pH 7.5, 20% PEG 3350) and H9 (200 m*M* sodium/potassium tartrate, 100 m*M* Bis-Tris propane pH 8.5, 20% PEG 3350). Crystals were cryoprotected in a buffer consisting of well solution to which 20% ethylene glycol was added.

A native data set was collected in-house at Cu *K*α wavelength using a Rigaku SuperBright FR-E^+^ rotating-anode X-ray generator equipped with Osmic VariMax HF optics and a Saturn 944+ CCD detector (Table 1 ▶, native Cu *K*α). The diffraction data were reduced with *XDS*/*XSCALE* (Kabsch, 2010 ▶) and scaled to 1.85 Å resolution in space group *C*222_1_.

In order to obtain experimental phase information, a crystal from the same well was incubated for 5 min in an iodide-containing buffer consisting of 200 m*M* sodium/potassium tartrate, 100 m*M* HEPES pH 7.0, 25% PEG 3350, 250 m*M* KI. A data set with high multiplicity was collected in-house using the same set up as described above. 720 images were collected with a width per frame of 0.5°. The diffraction data were reduced with *XDS*/*XSCALE* (Kabsch, 2010 ▶) and scaled to 2.0 Å resolution. Data analysis in *XSCALE* (Anomalous Correlation and SigAno) indicated a strong anomalous signal out to 2.0 Å resolution.

A high-resolution data set was collected on beamline 5.0.1 of the Berkeley Center for Structural Biology in the context of the Collaborative Crystallography program. The wavelength of this beamline is optimized for an Se-SAD experiment: 0.9774 Å/12.4 keV (Morton *et al.*, 2007 ▶). The beamline is equipped with a 3 × 3 tiled ADSC Q315r detector. The diffraction data were reduced with *XDS*/*XSCALE* (Kabsch, 2010 ▶) and scaled to 1.27 Å resolution. A weak anomalous signal can be detected in the *XSCALE* data analysis.

In order to validate ligand binding, the protein was incubated with 1 m*M* AMPPNP or GMPPNP and 2 m*M* MgCl_2_. A small optimization screen was set up: 15–20% PEG 3350, 200 m*M* sodium/potassium tartrate, 100 m*M* Bis-Tris pH 7.0–8.0. After crystals had grown to full size within a few days, they were incubated for 1 min in a cryoprotection buffer consisting of 1 m*M* AMPPNP or GMPPNP, 2 m*M* MgCl_2_, 15% ethylene glycol, 21% PEG 3350, 165 m*M* sodium/potassium tartrate, 83 m*M* Bis-Tris pH 7.5. For each of the co-crystals, a data set with high multiplicity was collected at the home source as described for the iodide soak above. Each data set was scaled to 1.7 Å resolution. For another AMPPNP cocrystal from the same optimization screen, a data set with high multiplicity was collected at a wavelength of 1.3476 Å on beamline 5.0.2 of the Berkeley Center for Structural Biology in the context of the Collaborative Crystallography program. The wavelength was selected to be at lower energy than the Zn edge (1.284 Å/9.659 keV) but at significantly higher energy than the Mn edge (1.896 Å/6.359 keV) The ALS beamline is equipped with a 3 × 3 tiled ADSC Q315r detector. The data were scaled to 1.6 Å resolution.

### 2.3. Structure solution and refinement

The packing density of the crystals (Matthews, 1968 ▶), *V*_M_ = 2.2 Å^3^ Da^−1^ (44% solvent content), suggested one molecule of BrabA.11339.a per asymmetric unit. A search of the Protein Data Bank with *BLAST* (Altschul *et al.*, 1997 ▶) did not yield any structural homologs beyond 23% sequence identity. Attempts to solve the structure of BrabA.11339.a by molecular replacement with *Phaser* (McCoy *et al.*, 2007 ▶) using the initial native data set (native Cu *K*α, 1.85 Å resolution) and PDB entry 3g1p (23% identity; K. Podzelinska, S. He, M. Wathier, A. Yakunin, M. Proudfoot, B. Hove-Jensen, D. Zechel & Z. Jia, unpublished work) as search models were not successful. Experimental phases were obtained independently from both the iodide soak and the high-resolution synchrotron data set.

#### 2.3.1. Iodide data set

Data analysis with *XSCALE* indicated a strong anomalous signal to 2.0 Å resolution. *phenix.hyss* (Grosse-Kunstleve & Adams, 2003 ▶) was used to locate 11 anomalous scatterers in the data set from the iodide soak. The anomalous sub­structure was refined with *Phaser* (McCoy *et al.*, 2007 ▶), which then extended the anomalous substructure to 16 sites and yielded a figure of merit (FOM) of 0.49 to 2.0 Å resolution. The experimental phases obtained from *Phaser* were improved with the *CCP*4 program *Parrot* (Cowtan, 2010 ▶). An initial model (256 residues, *R*_work_ = 0.307) was built with *ARP*/*wARP* (Langer *et al.*, 2008 ▶), followed by iterative rounds of manual real-space fitting in *Coot* (Emsley *et al.*, 2010 ▶) and reciprocal-space refinement with *REFMAC*5 (Murshudov *et al.*, 2011 ▶).

#### 2.3.2. Native synchrotron data set

In a separate approach, the native high-resolution synchrotron data set was used to phase the BrabA.11339.a structure. The *XSCALE* analysis had indicated a moderate anomalous signal to ∼2.0 Å resolution. *phenix.hyss* (Grosse-Kunstleve & Adams, 2003 ▶) was able to locate two strong anomalous scatterers when the diffraction data were limited to 3 Å resolution. *Phaser* (McCoy *et al.*, 2007 ▶) refined and extended the anomalous substructure to three atoms and yielded an FOM of 0.360 using data to 1.8 Å resolution. After density improvement with *Parrot* (Cowtan, 2010 ▶), *ARP*/*wARP* (Langer *et al.*, 2008 ▶) could build a good initial model with 267 residues and a *R*_work_ of 0.200 with data to 1.7 Å resolution.

#### 

The model for the high-resolution structure of BrabA.11339.a contains one protein molecule spanning residues Ser3–Val272, two manganese ions, a potassium ion, a sodium ion, a guanosine 5-­monophosphate (GMP), a tartrate molecule and 348 water molecules. The final structure was validated with *Coot* (Cowtan, 2010 ▶) and *MolProbity* (Chen *et al.*, 2010 ▶). The structure was deposited in the PDB with the identifier 3md7 (see Table 2 ▶).

#### 

The three structures cocrystallized with AMPPNP or GMPPNP were solved by direct refinement of the high-resolution structure from which all nonprotein atoms had been removed. Data and refinement statistics are summarized in Tables 1 ▶ and 2 ▶.

## 3. Results and discussion

The target BrabA.11339.a entered the SSGCID pipeline as a ‘β-­lactamase-like’ protein from *B. melitensis*. A search of sequence databases for homologies yielded a variety of annotations, primarily other ‘β-lactamase-like’ proteins, metal-dependent hydrolases, un­characterized proteins, PhnP (a phosphodiesterase of the carbon–phosphorus lyase pathway for phosphonate degradation) proteins and tRNase Z proteins. However, the sequence is highly conserved between various species, with many proteins sharing more than 50% sequence identity. A search of the Protein Data Base yielded a striking lack of structures with high sequence homology. The phosphodiesterase of the carbon–phosphorus lyase pathway (PhnP; PDB entry 3g1p) from *E. coli* is the closest homologue; PhnP shares 23% sequence identity with BrabA.11339.a, with a large number of inserts and deletions.

BrabA.11339.a readily crystallized in a well diffracting *C*-centered orthorhombic crystal form. The structure could not be solved by molecular replacement. SSGCID’s first approach to obtaining experimental phases is quick soaks with iodide (Abendroth *et al.*, 2010 ▶, 2011 ▶), following the ideas of Nagem and Dauter (Nagem *et al.*, 2001 ▶). The principles behind this approach are (i) the strong anomalous signal of iodide at Cu *K*α X-ray wavelength (*f*′′ = 6.8; Bricogne *et al.*, 2003 ▶), (ii) the compatibility of iodide with weak binding sites of different natures, both hydrophobic and basic, and (iii) the high concentrations of iodide that a crystal lattice can tolerate. In the SSGCID context, this experimental phasing strategy has been extremely successful (Abendroth *et al.*, 2010 ▶, 2011 ▶). For BrabA.11339.a the phasing information was not only obtained from iodide ions: two metal ions in the active site and a potassium ion contributed in addition to the iodide ions. In fact, the high-resolution native data set was collected on a beamline which was optimized for data collection at the Se edge (0.9774 Å). The minor anomalous signal from these three anomalous scatterers was strong enough to phase the structure independently. The structure solution of BrabA.11339.a underlines the power of anomalous phasing by both intrinsic anomalous scatterers such as metals and externally added scatterers such as iodide.

**Figure 1:**
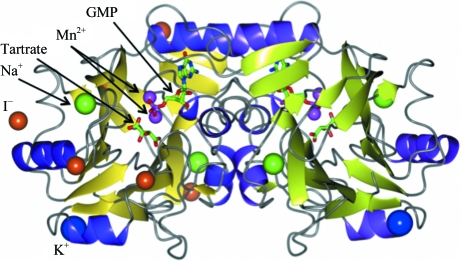
Structure of BrabA.11339.a dimer. In the ribbon representation of the crystallo­graphic dimer the β-strands for each protomer are colored slightly differently. The crystallographic dyad runs vertically. Various ions are shown as spheres: Mn^2+^ in magenta, K^+^ in blue and Na^+^ in green. Five strong iodide sites are shown as orange spheres for one protomer. GMP and tartrate are shown as stick models.

**Figure 2:**
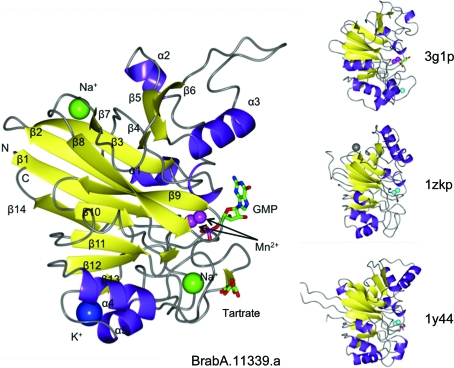
Fold comparison. Comparison of the fold of BrabA.11339.a with its structural homologues. 3g1p is the PhnP protein from *E. coli*, 1zkp is a putative ribonuclease from *B. anthracis* and 1y44 is ribonuclease Z from *B. subtilis*. The structural homology is focused around the two β-sheets and helices α4 and α5. Color scheme: Mn, magenta; Zn, cyan; Na, green; K, blue

BrabA.11339.a crystallizes with one monomer per asymmetric unit. A dimer is formed by crystallographic symmetry (Fig. 1 ▶). The dimer buries 1444 Å^2^ accessible surface per protomer in the interface as calculated by *PISA* (Krissinel & Henrick, 1997 ▶). BrabA.11339.a assumes a metallo-β-lactamase-like fold with two central β-sheets (Fig. 2 ▶). A search for structural homology using *SSM* (Krissinel & Henrick, 2004 ▶) reveals as the closest structural homologs the PhnP protein (PDB entry 3g1p, 1.7 Å r.m.s.d., 25% sequence identity), a putative ribonuclease (PDB entry 1zkp, 1.9 Å r.m.s.d., 21% sequence identity; Midwest Center for Structural Genomics, unpublished work) and ribonuclease Z (PDB entry 1y44, 2.3 Å r.m.s.d., 21% sequence identity; de la Sierra-Gallay *et al.*, 2005 ▶) (see Fig. 2 ▶). The structural homology is focused mostly around the central β-sheets and the helices α4 and α5, while there is more structural variety between β-­strands β5 and β6.

**Figure 3:**
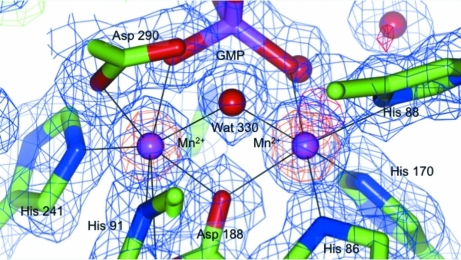
Dinuclear manganese center. The active site of BrabA.11339.a consists of two manganese ions (magenta), which are bridged by a water molecule (red). The phosphate group of GMP is in close proximity to this activated water molecule. The σ_A_-weighted 2*F*_o_ − *F*_c_ electron density for the high-resolution data set is contoured at 1σ (blue) and the corresponding *F*_o_ − *F*_c_ electron density is contoured at ±3σ (green/red). The anomalous Fourier electron density is contoured at 10σ (orange). The strong anomalous Fourier density at all three wavelengths indentifies the active-site metal as manganese. For bond distances refer to Table 3 ▶; for anomalous peak heights refer to Table 4 ▶.

As a very typical feature of β-lactamase-like proteins and the phosphodiesterase protein family, a two-metal center is present in the active site of BrabA.11339.a (Fig. 3 ▶). The two metals are 3.40 Å apart, bridged by water 330 and liganded by several histidine residues (His86, His88, His170 and His241) with bond distances between 2.22 and 2.24 Å, aspartate residues (Asp90 and Asp188) with bond distances between 2.28 and 2.31 Å and the phosphate group of GMP. The bond distances around the metal center are summarized in Table 3 ▶. They fall within the distances expected for the coordination of manganese ions (Zheng *et al.*, 2008 ▶).

Podzelinska *et al.* (2009 ▶) have performed a detailed functional analysis of the metal-dependency of PhnP; they report a strong preference for Mn^2+^ and Ni^2+^ (Podzelinska *et al.*, 2009 ▶) and model the metals as manganese ions in their structure. In the 1zkp structure of a putative ribonuclease (Midwest Center for Structural Genomics, unpublsihed work) the metals are modeled as Zn^2+^ ions, likely because of the presence of zinc chloride in the crystallization buffer. In the structure of RNAse Z (PDB entry 1y44) the metals are modeled as Zn^2+^ ions because of functional studies, even though this is not further discussed in the structure paper and the *B* factors of the metal ions are higher than for the protein environment (de la Sierra-Gallay *et al.*, 2005 ▶).

The iodide data set already indicated significant anomalous density for the two active-site metals and the potassium site. In order to analyze the metal center in more detail, diffraction data sets with high multiplicity were collected at long X-ray wavelengths from the ligand co-crystals: in-house at λ = 1.5418 Å and at the synchrotron at λ = 1.3476 Å. Anomalous Fourier peak heights for the metal sites are summarized in Table 4 ▶(*a*) and are compared with the theoretical *f*′′ coefficients for various atoms in Table 4 ▶(*b*). Potassium was part of the crystallization, soaking and cryobuffer at high concentrations that were consistent between the data sets. The occupancy of the potassium ion can therefore assumed to be constant and can be considered as a reference. Interestingly, the potassium-ion site is not conserved between the structural homologs. For both long-wavelength data sets and the Se-edge data set strong anomalous Fourier peaks can be observed for the two active-site metals. Each of the peaks is about four times as strong as the signal for potassium. Manganese is the only metal in question that has a significant anomalous signal at all three wavelengths. The anomalous signal for Zn^2+^ is strong at the Se edge, but is very weak at either of the longer wavelengths. The anomalous signal for Ni^2+^ is strong at the Se edge and at 1.3476 Å; however, it is low at 1.5418 Å. The bond distances between the metals are in the range expected for Mn^2+^ ions (Zheng *et al.*, 2008 ▶). Therefore, the dinuclear center of BrabA.11339.a can be assigned as two manganese ions with a high degree of confidence.

**Figure 4:**
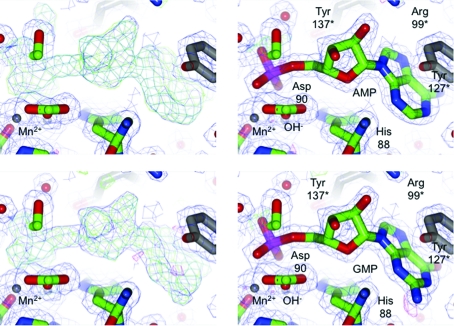
AMP-bound and GMP-bound structures. BrabA.11339.a was cocrystallized with AMPPNP and GMPPNP, variants of AMP or GMP that are nonhydrolyzable between the β-­phosphate and γ-phosphate. Left panels, OMIT densities for the AMPPNP (top) and GMPPNP (bottom) cocrystals (see Fig. 3 ▶ for colors). The σ_A_-weighted 2*F*_o_ − *F*_c_ electron density for the high-resolution data set is contoured at 1σ (blue) and the corresponding *F*_o_ − *F*_c_ electron density is contoured at ±3σ (green/red). The right panels show the refined densities for the models refined with AMP (top) and GMP (bottom), respectively. No density is visible beyond the α-phosphate. It is likely that BrabA.11339.a has hydrolyzed the β-phosphate. The nucleotides are bound in the dimer interface and engage in multiple interactions with both protomers.

In the native structure, a partially occupied GMP molecule can be observed in the immediate proximity of the binuclear Mn^2+^ center. At no stage during the purification was GMP added to the protein; thus, it must have been copurified from the expression host. In order to confirm the binding of nucleotides, crystals were grown in the presence of GMPPNP and AMPPNP. In both cases, however, very solid electron density could be observed for GMP and AMP, respectively (see Fig. 4 ▶). The α-phosphate group is very well defined, while there is no density for the β-phosphate or γ-phosphate groups. The γ-­phosphate groups of GMPPNP or AMPPNP, which had been added during crystal growth and had been supplemented for a short soak in the cryobuffer, are nonhydrolyzable; the β-phosphate groups, however, can be hydrolyzed. It can be assumed that BrabA.11339.a is able to hydrolyze AMPPNP and GMPPNP between the α-phosphate and the β-phosphate to AMP or GMP, respectively.

The GMP or AMP molecules are bound in the dimer interface and make multiple interactions. The phosphate group is involved in hydrogen bonds to both manganese ions, the bridging water molecule 330 (called water 1 in the ligand-bound structures), histidine residues His88 and His241, aspartate residue Asp90 and further water molecules (315, 454 and 511 in the high-resolution structure). The ribose group makes two interactions with the carboxyl group of Asp96 (O2* and O3*) and two interactions with the guanidine group of Arg99 (O2*), each from the other protomer. The guanine group stacks with Tyr127 from the other protomer. The guanine moiety engages in hydrogen bonds to the amide N atom of Ala89 (N3) and to water molecules. Neither of these interactions should be able to provide specificity for either AMP or GMP.

In the PhnP structure (PDB entry 3g1p), a malonate is modelled in the space that is occupied by the phosphate and the ribose. No ligand was modelled in the putative ribonuclease (PDB entry 1zkp). In the structure of ribonuclease Z from *B. subtilis* (PDB entry 1y44, r.m.s.d. 2.3–2.4 Å, 20% sequence identity) a phosphate ion is modelled in an identical location to the phosphate group of GMP in BrabA.11339.a, even though the *B* factors for the phosphate ion are significantly higher than those for the environment (de la Sierra-Gallay *et al.*, 2005 ▶). None of these three homologous structures are able to accommodate a GMP or an AMP molecule in the conformation observed in BrabA.11339.a without conformational changes.

The discovery of a purine monophosphate in the vicinity of the dinuclear manganese center allows speculation regarding the function of BrabA.11339.a. For various enzymes with a dinuclear center it is known that the bridging water is activated by the two metal ions. The water reacts as a hydroxide, starting a hydrolytic reaction. The phosphate group of the GMP or AMP is located in close proximity to this deprotonated water molecule 303 (2.2 Å). There is enough space in the active site to fit two more phosphate groups. It therefore seems likely that BrabA.11339.a is a GTP/ATP or GDP/ADP hydrolase.

The genetic context of BrabA.11339.a (BAB1_1016) hints at a function in nucleotide processing. Its genetic neighbors are BAB1_1012, DNA polymerase II subunit Δ; BAB1_1013, a hypothetical protein; BAB1_1014, metG, a methionyl-tRNA synthetase; BAB_1015, a TatD-related deoxyribonuclease; BAB1_1016, BrabA.11339.a; BAB1-1017, a cyclic β-1,2-glucan ABC transporter; BAB1_1018, a hypothetical protein; and BAB1_1019, an RNA-binding S4:pseudo­uridine synthase.

The structure of BrabA.11339.a and its binuclear manganese center were determined with high accuracy using high-resolution data and anomalous diffraction. The initially serendipitous discovery of GMP in the active site and the subsequent high-resolution co-crystal structures with *in situ* hydrolyzed AMP or GMP shed light on the function of this protein, which entered the SSGCID pipeline as a ‘β-­lactamase-like protein’. Further analysis will be needed to validate the biological function of this protein and its suitability for structure-guided drug design against the pathogen *B. melitensis*.

## Supplementary Material

PDB reference: BrabA.11339, 3md7

PDB reference: GMP complex, 3py5

PDB reference: 3py6

PDB reference: AMP complex, 3qh8

## Figures and Tables

**Table 1:** Data-collection statistics Values in parentheses are for the highest of 20 resolution shells.

Ligand	Iodide	Native Cu *K*α	Native ALS 501	GMP Cu *K*α	AMP Cu *K*α	AMP ALS 502
Wavelength (Å)	1.5418	1.5418	0.9774	1.5418	1.5418	1.3476
Space group	*C*222_1_	*C*222_1_	*C*222_1_	*C*222_1_	*C*222_1_	*C*222_1_
Unit-cell parameters (Å)	*a* = 72.71, *b* = 74.95, *c* = 98.27	*a* = 72.63, *b* = 75.04, *c* = 98.11	*a* = 72.89, *b* = 75.25, *c* = 98.36	*a* = 72.86, *b* = 75.23, *c* = 98.54	*a* = 72.85, *b* = 75.20, *c* = 98.53	*a* = 73.21, *b* = 75.53, *c* = 98.94
Resolution range (Å)	50–2.00 (2.05–2.00)	50–1.85 (1.90–1.85)	50–1.27 (1.30–1.27)	50–1.70 (1.74–1.70)	50–1.70 (1.74–1.70)	50–1.60 (1.64–1.60)
Unique reflections	17854 (1257)	23011 (1520)	71257 (5123)	29984 (2068)	29964 (2069)	36362 (2574)
Multiplicity	12.4 (6.9)	5.4 (1.90)	9.5 (6.7)	9.1 (4.4)	9.1 (4.5)	12.4 (6.1)
Completeness (%)	99.3 (94.3)	98.9 (91.3)	99.7 (97.2)	99.5 (93.1)	99.5 (93.6)	99.6 (95.9)
*R*_merge_†	0.072 (0.140)	0.048 (0.149)	0.051 (0.489)	0.037 (0.147)	0.041 (0.203)	0.069 (0.390)
Mean *I*/σ(*I*)	27.1 (11.4)	24.7 (5.1)	28.3 (3.9)	40.0 (9.2)	35.2 (7.7)	25.1 (5.8)

† *R*_merge_ = 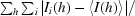 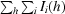.

**Table 2:** Refinement and model statistics The models for the iodide and native Cu *K*α data sets have only been partially refined. Preliminary refinement statistics are shown in italics. Values in parentheses are for the highest of 20 resolution shells.

Ligand	Iodide	Native Cu *K*α	Native ALS 501	GMP Cu *K*α	AMP Cu *K*α	AMP ALS 502
Resolution range (Å)	50–2.00 (2.05–2.00)	50–1.85 (1.90–1.85)	50–1.27 (1.30–1.27)	50–1.70 (1.74–1.70)	50–1.70 (1.74–1.70)	50–1.60 (1.64–1.60)
*R*_cryst_†	*0.242 (0.226)*	*0.181 (0.213)*	0.107 (0.152)	0.140 (0.163)	0.164 (0.174)	0.144 (0.223)
*R*_free_†	*0.295 (0.281)*	*0.228 (0.266)*	0.134 (0.203)	0.168 (0.206)	0.196 (0.211)	0.168 (0.260)
R.m.s.d. bonds (Å)	*0.017*	*0.023*	0.018	0.013	0.015	0.015
R.m.s.d. angles (°)	*1.65*	*1.94*	1.87	1.52	1.64	1.65
Protein atoms			2116	2116	2110	2118
Nonprotein atoms			380	381	344	344
Mean *B* factor (Å^2^)			9.85	8.13	10.4	10.6
Residues in favored region			243 [98%]	251 [97%]	248 [96%]	253 [98%]
Residues in allowed region			4 [1.4%]	6 [2.3%]	9 [3.5%]	5 [1.9%]
Residues in disallowed region			1 [0.4%]	1 [0.4%]	1 [0.4%]	1 [0.4%]
*MolProbity* score [percentile]			1.92 [92nd]	1.27 [98th]	1.38 [96th]	1.08 [99th]
PDB code	n/a	n/a	3md7	3py6	3py5	3qh8

† *R*_cryst_ = 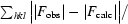 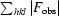. The free *R* factor was calculated with an equivalent equation using 5% of the reflections that were omitted from the refinement.

**Table 3:** Bond distances of the dinuclear Mn^2+^ center for the high-resolution data set

Atom	Distance from Mn300 (Å)	Atom	Distance from Mn301 (Å)
Mn301	3.40	Mn300	3.40
Wat330	2.10	Wat330	2.07
GMP O1P	2.38	GMP O3P	2.20
Asp90 OD2	2.28		
Asp188 OD2	2.28	Asp188 OD2	2.31
Asp188 OD1	3.00		
His241 NE2	2.23	His86 NE2	2.24
		His88 ND1	2.22
		His170 NE2	2.22

**(a):** Anomalous Fourier maps were calculated over the full resolution range for all three data sets. Peaks were found with the *CCP*4 program *PEAKMAX*.

Atom	0.9774 Å	1.3476 Å	Cu *K*α AMP	Cu *K*α GMP
Mn300	33.8	31.8	36.6	45.8
Mn301	42.4	42.1	40.5	47.2
K302	8.8	12.6	11.7	14.3
Cys15 SG	6.4	5.7	7.2	9.0
Cys31 SG	6.5	4.9	8.3	10.0
Met71 SD	5.7	8.3	9.2	9.9
Met109 SD	6.7	6.6	7.3	7.9
Cys128 SG	5.3	5.1	8.0	10.3
Met143 SD	5.0	6.0	5.7	5.4
Cys186 SG	6.5	5.5	7.5	9.3
Met242 SD	6.1	7.3	8.3	10.4
Met252 SD	—	—	5.9	5.6

**(b):** Anomalous Fourier coefficients *f*′′ (e^−^) for selected elements at Cu *K*α wavelength and 12.4 keV are listed for comparison (Bricogne *et al.*, 2003 ▶). Manganese is the only metal in question that has a stronger signal than potassium at both wavelengths.

	0.9774 Å	1.3476 Å	1.5418 Å
I	3.18	5.49	6.83
S	0.23	0.43	0.56
Mn	1.30	2.26	2.80
Zn	2.48	0.55	0.71
K	0.46	0.84	1.08
Ni	1.95	3.30	0.51

## References

[bb1] Abendroth, J., Edwards, T. E., Staker, B. L., Myler, P. J. & Stewart, L. J. (2010). Northwest Crystallography Workshop, University of British Columbia, Canada.

[bb2] Abendroth, J., Gardberg, A. S., Robinson, J. I., Christensen, J. S., Staker, B. L., Myler, P. J., Stewart, L. J. & Edwards, T. E. (2011). *J. Struct. Funct. Genomics*, **12**, 83–95. 10.1007/s10969-011-9101-7 PMC3123459 21359836

[bb3] Altschul, S. F., Madden, T. L., Schäffer, A. A., Zhang, J., Zhang, Z., Miller, W. & Lipman, D. J. (1997). *Nucleic Acids Res.* **25**, 3389–3402. 10.1093/nar/25.17.3389 PMC146917 9254694

[bb4] Aslanidis, C. & de Jong, P. J. (1990). *Nucleic Acid Res.* **18**, 6069–6074. 10.1093/nar/18.20.6069 PMC332407 2235490

[bb5] Bricogne, G., Vonrhein, C., Flensburg, C., Schiltz, M. & Paciorek, W. (2003). *Acta Cryst.* D**59**, 2023–2030. 10.1107/s0907444903017694 14573958

[bb6] Chen, V. B., Arendall, W. B., Headd, J. J., Keedy, D. A., Immormino, R. M., Kapral, G. J., Murray, L. W., Richardson, J. S. & Richardson, D. C. (2010). *Acta Cryst.* D**66**, 12–21. 10.1107/S0907444909042073 PMC2803126 20057044

[bb7] Corbel, M. J. (1997). *Emerg. Infect. Dis.* **3**, 213–221. 10.3201/eid0302.970219 PMC2627605 9204307

[bb8] Cowtan, K. (2010). *Acta Cryst.* D**66**, 470–478. 10.1107/S090744490903947X PMC2852311 20383000

[bb9] Emsley, P., Lohkamp, B., Scott, W. G. & Cowtan, K. (2010). *Acta Cryst.* D**66**, 486–501. 10.1107/S0907444910007493 PMC2852313 20383002

[bb10] Grosse-Kunstleve, R. W. & Adams, P. D. (2003). *Acta Cryst.* D**59**, 1966–1973. 10.1107/s0907444903018043 14573951

[bb11] Kabsch, W. (2010). *Acta Cryst.* D**66**, 125–132. 10.1107/S0907444909047337 PMC2815665 20124692

[bb12] Krissinel, E. & Henrick, K. (1997). *J. Mol. Biol.* **372**, 774–797. 10.1016/j.jmb.2007.05.022 17681537

[bb13] Krissinel, E. & Henrick, K. (2004). *Acta Cryst.* D**60**, 2256–2268. 10.1107/S0907444904026460 15572779

[bb14] Langer, G., Cohen, S. X., Lamzin, V. S. & Perrakis, A. (2008). *Nature Protoc.* **3**, 1171–1179. 10.1038/nprot.2008.91 PMC2582149 18600222

[bb15] Matthews, B. W. (1968). *J. Mol. Biol.* **33**, 491–497. 10.1016/0022-2836(68)90205-2 5700707

[bb16] McCoy, A. J., Grosse-Kunstleve, R. W., Adams, P. D., Winn, M. D., Storoni, L. C. & Read, R. J. (2007). *J. Appl. Cryst.* **40**, 658–674. 10.1107/S0021889807021206 PMC2483472 19461840

[bb17] Mehlin, C. *et al.* (2006). *Mol. Biochem. Parasitol.* **148**, 144–160. 10.1016/j.molbiopara.2006.03.011 16644028

[bb18] Moreno, E. & Moriyon, I. (2002). *Proc. Natl Acad. Sci. USA*, **99**, 1–3. 10.1073/pnas.022622699 PMC117501 11782541

[bb19] Morton, S., Glossinger, J., Smith-Baumann, A., McKean, J. P., Trame, C., Dickert, J., Rozales, A., Dauz, A., Taylor, J., Zwart, P., Duarte, R., Padmore, H., McDermott, J. & Adams, P. (2007). *Synchrotron Radiat. News*, **20**(4), 23–30.

[bb20] Murshudov, G. N., Skubák, P., Lebedev, A. A., Pannu, N. S., Steiner, R. A., Nicholls, R. A., Winn, M. D., Long, F. & Vagin, A. A. (2011). *Acta Cryst.* D**67**, 355–367. 10.1107/S0907444911001314 PMC3069751 21460454

[bb21] Nagem, R. A. P., Dauter, Z. & Polikarpov, I. (2001). *Acta Cryst.* D**57**, 996–1002. 10.1107/s0907444901007260 11418768

[bb22] Newman, J., Egan, D., Walter, T. S., Meged, R., Berry, I., Ben Jelloul, M., Sussman, J. L., Stuart, D. I. & Perrakis, A. (2005). *Acta Cryst.* D**61**, 1426–1431. 10.1107/S0907444905024984 16204897

[bb23] Podzelinska, K., He, S.-M., Wathier, M., Yakunin, A., Proudfoot, M., Hove-Jensen, B., Zechel, D. L. & Jia, Z. (2009). *J. Biol. Chem.* **284**, 17216–17226. 10.1074/jbc.M808392200 PMC2719359 19366688

[bb24] Sierra-Gallay, I. L. de la, Pellegrini, O. & Condon, C. (2005). *Nature (London)*, **433**, 657–661. 10.1038/nature03284 15654328

[bb25] Studier, F. W. (2005). *Protein Expr. Purif.* **41**, 207–234. 10.1016/j.pep.2005.01.016 15915565

[bb26] Young, E. J. (1995). *Clin. Infect. Dis.* **21**, 283–289.

[bb27] Zheng, H., Chruszcz, M., Lasota, P., Lebioda, L. & Minor, W. (2008). *J. Inorg. Biochem.* **102**, 1765–1776. 10.1016/j.jinorgbio.2008.05.006 PMC2872550 18614239

